# Strong inhibition of xanthine oxidase and elastase of *Baccharis trimera* (Less.) DC stem extract and analysis of biologically active constituents

**DOI:** 10.3389/fphar.2023.1160330

**Published:** 2023-05-25

**Authors:** Seung-Yub Song, Sung-Ho Lee, Min-Suk Bae, Dae-Hun Park, Seung-Sik Cho

**Affiliations:** ^1^ Department of Pharmacy, College of Pharmacy, Mokpo National University, Muan, Jeonnam, Republic of Korea; ^2^ Department of Biomedicine, Health, and Life Convergence Sciences, BK21 Four, Biomedical and Healthcare Research Institute, Mokpo National University, Muan, Jeonnam, Republic of Korea; ^3^ Department of Environmental Engineering, Mokpo National University, Muan, Jeonnam, Republic of Korea; ^4^ College of Oriental Medicine, Dongshin University, Naju-si, Jeonnam, Republic of Korea

**Keywords:** *Baccharis trimera*, xanthine oxidase, elastase, hyperuricemia, anti-wrikle

## Abstract

**Introduction:** In the present study, strong xanthine oxidase and elastase activities of *Baccharis trimera* (Less) DC stem (BT) were evaluated and active ingredients were identified to determine the possibility of using BT extract as an anti-hyperuricemia (gout) and cosmetic functional material.

**Methods:** Hot water, 20, 40, 60, 80, and 100% ethanolic extracts of BT were prepared. The hot water extract had the highest extraction yield whereas the 100% ethanolic extract had the lowest yield.

**Results and discussion:** Antioxidant effects were investigated based on DPPH radical scavenging activity, reducing power, and total phenolic contents. The 80% ethanolic extract showed the highest antioxidant activity. However, the 100% ethanol BT extract showed strong xanthine oxidase and elastase inhibitory activities. Functional substances were thought to be caffeic acid and luteolin. Minor active substances such as o-coumaric acid, palmitic acid, naringenin, protocatechoic acid, and linoleic acid were identified. Through this study, we firstly reported evidence that BT stem extract could be used as functional materials with anti-hyperuricemia and skin disease improving effects. BT stem extract could be used as an anti-hyperuricemia (gout) natural drug or cosmetic material. For further study, practical studies such as optimizing BT extraction and functional experiments for hyperuricemia (gout) and skin wrinkle improvement are considered necessary.

## 1 Introduction

The popular use of medicinal plants has increased exponentially in recent decades. Since medicinal plants are the basis for the development of new drugs using natural medicines or single active substances, it is important to collect data on characteristics, active ingredients, and pharmacology of plants (Wu et al., 2020). However, there is still little scientific evidence to validate the use of these plants as curative or preventive of different diseases. A medicinal plant used in popular culture is BT that is widely distributed in South America In Brazil, this plant is popularly known as carqueja and used to treat diseases such as diabetes, inflammatory processes, and liver diseases (Abad and Bermejo, 2007). This plant possesses various biological effects such as hypoglycemic (Ximenes et al., 2022), hepatoprotective (Rabelo et al., 2018), antioxidant (Cruz Padua et al., 2013), and anti-inflammatory (De Oliveira et al., 2012).

Biological activities of BT are mainly known for their anti-inflammatory, hepatoprotective, and anti-diabetic activities. It has been reported to contain apigenin, 7,4′-di-O-methyl-apigenin, cirsimaritin, caffeic acid derivatives, eupatorin, genkwanin, hispidulin, isoquercetin, luteolin, nepetin, quercetin, 3-O-methylquercetin, 5,6-dihydroxy-7,3′,4′-trimethoxyflavone, and rutin (Soicke and Leng-Peschlow, 1987; Gené et al., 1996; Simões-Pires et al., 2005).

In the present study, we evaluated various biological activities of BT stem in addition to their traditional therapeutic uses and investigated the possibility of utilizing BT as medical and cosmetic materials. As a result of this study, BT ethanolic extract showed strong xanthine oxidase and elastase inhibiting activities. HPLC and GCMS confirmed biomarkers related to enzyme inhibition. Results of this study suggest that BT could be used as a potential material to treat inflammatory diseases related to gout besides its use for whitening and wrinkle improvement.

## 2 Materials and methods

### 2.1 Plant material and extract preparation

*Baccharis trimera* Stem was supplied by Missionario Evangelico Três Fronteira (Brazil). A voucher specimen (MNUCSS−BT−01) was deposited at Mokpo National University (Muan, Korea). Dried BT stem (20 g) was extracted twice with 100 mL of (20–100) % ethanol solution at RT for 48 h or extracted with boiled water for 4 h. The resultant solution was freeze dried for further experiments.

### 2.2 DPPH free radical assay

Antioxidant activity was determined following a DPPH radical scavenging assay. Briefly, sample solution was added to DPPH solution (0.4 mM) and mixed. The mixture was allowed to react at room temperature (RT) for 10 min. This mixture was measured at 517 nm with a microplate reader (Perkin Elmer, Waltham, MA, United States) (Song et al., 2017).

### 2.3 Determination of total phenolic content

Total phenolic content was determined by Folin–Ciocalteu assay (Song et al., 2017). Samples were mixed with sodium carbonate solution and Folin–Ciocalteu phenol reagent for 10 min. Absorbance was measured at 750 nm and compared with a gallic acid calibration curve. Results are expressed as milligrams of gallic acid equivalents per Gram of sample.

### 2.4 Determination of xanthine oxidase (XO) inhibitory activity

Sample and xanthine (1 mM) were mixed in 0.1 M potassium phosphate buffer (pH 7.5) and reacted at room temperature for 5 min. After adding xanthine oxidase (0.1 unit/mL) to the reaction solution, the reaction was carried out at 37°C for 15 min. Then 1 N HCl was added to stop the reaction. Centrifugation was performed at 15,000 rpm for 10 min. The supernatant was separated and measured at 292 nm. We calculated the enzyme activity as the ratio (%) of inhibitory activity compared to the control (Song et al., 2022).

### 2.5 Determination of elastase inhibitory activity

The assay was performed according to published protocols (Chiocchio et al., 2018). Briefly, elastase (10 ug/mL) was mixed with Tris–HCl (0.2 M) of STANA (2.5 mM, N-Succinyl-Ala-Ala-Ala-*p*-nitroanilide) and each sample at 37°C for 30 min. This mixture was centrifuged at 15,000 rpm for 10 min. The absorbance was measured at 405 nm.

### 2.6 Identification and quantitication of active constituents using GC-MS

Active constituents from BT were analyzed using GC−MS based on a previous report (Song et al., 2022). with slight modifications. Briefly, Agilent 7890 gas chromatography (GC) and Agilent 5975 quadrupole mass spectrometry (MS) system (Agilent Technologies, Palo Alto, CA, United States) were utilized to analyze molecular mass fragments of (50–550 amu) of the BT extract. After using an Agilent HP−5MS fused silica capillary column (30 mm length × 0.25 mm i.d., 0.25 μm film thickness), mass fragments were ionized under electron ionization (EI) conditions. A GC oven was isothermally programmed at 65°C for 10 min at (10–300) min^−1^ with helium (He) as a carrier gas. All data were compared with the system library (NIST 2017).

### 2.7 Constituent profiling by high-performance liquid chromatography (HPLC) analysis

**FIGURE 1:**
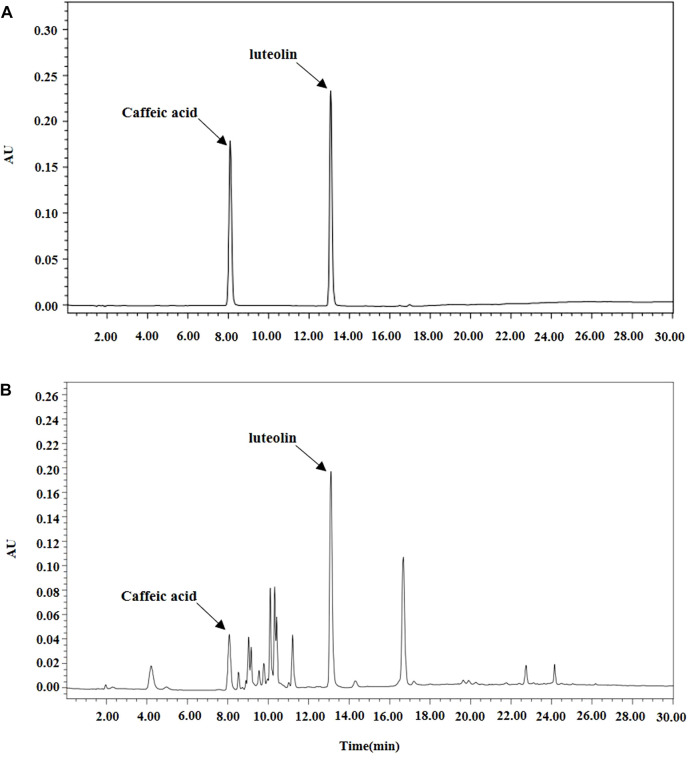
Analysis of Baccharis trimera extracts by High Performance Liquid Chromatography (HPLC). **(A)** Standard; **(B)** Sample extract.

Caffeic acid and luteolin (Sigma-Aldrich, United States) analyses of BT extracts were performed with HPLC. All HPLC analyses were performed using an Alliance 2695 HPLC system (Waters; Milford, MA, United States) equipped with a photodiode array detector. The analysis method was used by slightly modifying the previous analysis method and Figure 1 shows chromatographic profiles (Song et al., 2018).

### 2.8 Statistical analysis

The data were analysised using Excel^®^ software. All experiments were performed in triplicate. Results were expressed as mean and standard deviation, and statistical significance was expressed as *p*-value after performing one way ANOVA.

## 3 Results

### 3.1 Extraction yields and analysis of active compounds

Table 1 shows the yield of BT extract. The hot water extract showed the highest yield at 13.24%, while the 100% ethanol extract showed the lowest BT extraction yield at 5.56%.

**TABLE 1:** Extraction yields of Baccharis trimera extracts.

Extract	Extraction (%, w/w)
Hot Water	13.24
20% EtOH	9.28
40% EtOH	10.55
60% EtOH	10.05
80% EtOH	11.02
100% EtOH	5.56

As shown in Figure 1, caffeic acid and luteolin were identified as active substances. Table 2 shows analysis results of the 100% ethanol extract by GCMS. O-coumaric acid, palmitic acid, naringenin, protocatechoic acid, and linoleic acid were found to be its main constituents.

**TABLE 2:** Gas chromatography-mass spectrometry (GC-MS) analyses of Baccharis trimera extracts.

RT (min)	Hit name	Quality	M.W.	Composition (%)
27.943	Caffeic acid, 3TMS derivative	99	396	4.21
24.682	o-Coumaric acid, 2TMS derivative	99	308	3.66
27.091	Palmitic Acid, TMS derivative	99	328	2.23
17.792	Glycerol, 3TMS derivative	91	308	1.43
33.425	(.+/−.)-Naringenin, O,O′-bis(trimethylsilyl)-	99	416	1.09
24.785	Protocatechoic acid, 3TMS derivative	94	370	1.07
28.601	9(E),11(E)-Conjugated linoleic acid	96	352	1.03

### 3.2 Antioxidant effects of BT extracts

As a result of evaluating the electron donating ability of BT extract with ascorbic acid at the same concentration (25 μg/mL), the 80% ethanolic extract (84%) showed an activity similar to ascorbic acid (79%). Reducing power and total phenol contents of 80% ethanolic extract were the lowest at 34.33 as ascorbic acid eq. μg/100 μg extract and 73.42 as gallic acid eq. mg/g, respectively (Table 3). Eighty percent of BT extract showed the highest DPPH radical scavenging effect, reducing power and total phenol contents (88.12 as ascorbic Acid eq. μg/100 μg extract, 134.46 as gallic acid eq. mg/g).

**TABLE 3:** Antioxidant activities of *Baccharis trimera* extracts.

Extract	DPPH radical scavenging (%)	Reducing power (ascorbic acid eq. μg/100 μg extract)	Total phenolic content (gallic acid eq. Mg/g)
Vitamin C	79.75 ± 4.129^**^		—
Hot Water	39.95 ± 0.357^**,##^	45.77 ± 2.216^**^	74.80 ± 0.914^**^
20% EtOH	59.11 ± 3.418^**,##,++^	67.87 ± 3.527^**,##,++^	115.67 ± 6.726^**,##^
40% EtOH	63.63 ± 4.274^**,##,$^	75.80 ± 2.143^**,##,++^	128.42 ± 7.366^**,##^
60% EtOH	59.66 ± 3.535^**,##,++,@^	74.82 ± 4.352^**,##,++,$,@^	122.24 ± 1.918^**,##^
80% EtOH	84.27 ± 4.324^**,#,++,$$,@@,&&^	88.12 ± 4.018^**,#,+,$$,@^	134.46 ± 6.881^**,##,+,@^
100% EtOH	39.33 ± 1.221^**,##,$,&,%%^	34.33 ± 3.165^**,++,@,&&^	73.42 ± 6.400^**,+,$$,@@,&&^

Statistical significance was expressed as p-value. All experiments were repeated 3 times. For DPPH assay, p-valute were expressed as *p < 0.05 vs. CON; **p < 0.001 vs. CON; #p < 0.05 vs. Vit C; ##p < 0.001 vs. Vit C; +p < 0.05 vs. H.W; ++p < 0.001 vs. H.W; $p < 0.05 vs. 20% EtOH; $$p < 0.001 vs. 20% EtOH; @p < 0.05 vs. 40% EtOH; @@p < 0.001 vs. 40% EtOH; &p < 0.05 vs. 60% EtOH; &&p < 0.001 vs. 60% EtOH; %%p < 0.001 vs. 80% EtOH, For reducing Power and total Phenolic Content *p < 0.05 vs. CON; **p < 0.001 vs. CON; #p < 0.05 vs. H.W; ##p < 0.001 vs. H.W; +p < 0.05 vs. 20% EtOH; ++p < 0.001 vs. 20% EtOH; $p < 0.05 vs. 40% EtOH; $$p < 0.001 vs. 40% EtOH; @p < 0.05 vs. 60% EtOH; $$p < 0.001 vs. 60% EtOH; &p < 0.05 vs. 80% EtOH; &&p < 0.001 vs. 80% EtOH.

### 3.3 Xanthine oxidase inhibitory effects of BT extracts

**FIGURE 2:**
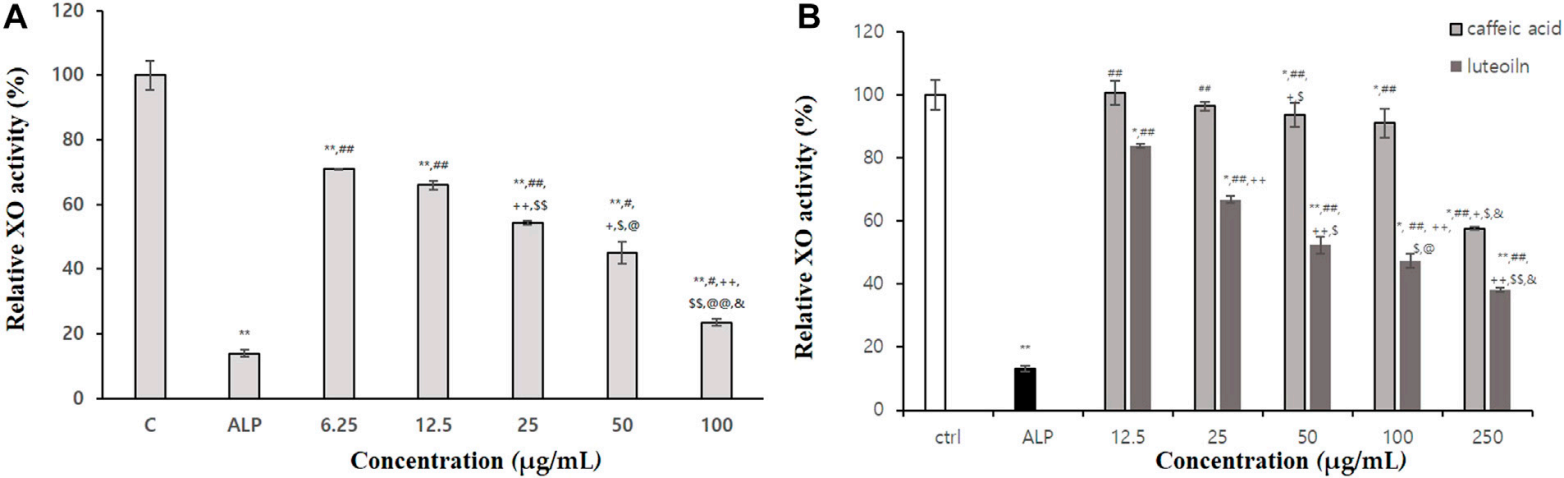
Xanthine oxidase (XO) inhibitory activities of 100% ethanolic extracts **(A)**, caffeic acid and luteolin **(B)**. Statistical significance was expressed as *p*-value. All experiments were repeated three times. In Fig A *p*-value were expressed as **p < 0.001 vs. *CON;* ^*#*^ *p < 0.05* vs. *allopueriol;* ^*##*^ *p < 0.001* vs. *allopueriol;* ^*+*^ *p < 0.05* vs. *6.25 μg/mL;* ^*++*^ *p < 0.001* vs. *6.25 μg/mL;* ^*$*^ *p < 0.05* vs. *25 μg/mL;* ^*$$*^ *p < 0.05* vs. *25 μg/mL;* ^*@*^ *p < 0.05* vs. *50 μg/mL;* ^*@* *@*^ *p < 0.001* vs. *50 μg/mL.* In Fig B, *p*-value were expressed as *p < 0.05 vs. CON; **p < 0.001 vs. *CON;* ^*##*^ *p < 0.001* vs. *allopueriol;* ^*+*^ *p < 0.05* vs. *12.5 μg/mL;* ^*++*^ *p < 0.001* vs. *12.5 μg/mL;* ^*$*^ *p < 0.05* vs. *25 μg/mL;* ^*@*^ *p < 0.05* vs. *50 μg/mL;* ^*&*^ *p < 0.05* vs. *100 μg/mL*.

As shown in Table 4, BT extract displayed excellent xanthine oxidase activity. Allopurinol, a control, when treated with 200 ug/mL, showed about 85.82% xanthine oxidase inhibitory activity. The 100% ethanol BT extract, when treated with 1 mg/mL, showed about 82.3% xanthine oxidase inhibitory activity. It was the most effective one among extracts. In Figure 2A, 100% BT extract concentration dependently inhibited xanthine oxidase. Caffeic acid and luteolin were among active substances that inhibited xanthine oxidase (42.4% and 61.7% inhibition at 250 μg/mL, Figure 2B). As a result of confirming the inhibitory effect, 100% ethanolic extract was found to be suitable as an anti-hyperuricemic herb medicine.

**TABLE 4:** Xanthine oxidase (XO) inhibitory activities of water and ethanolic extracts from *Baccharis trimera.*

Extract	Residual XO activity (%)
Allopurinol	4.67 ± 2.424^**^
Hot Water	50.20 ± 2.381^**,##^
20% EtOH	44.19 ± 4.612^**,##,+^
40% EtOH	45.67 ± 5.229^**,##,+^
60% EtOH	40.47 ± 6.450^**,##,+^
80% EtOH	43.14 ± 5.234^*,##,$,@,&^
100% EtOH	17.70 ± 3.712^**,#,++,$,@,&,%^

Statistical significance was expressed as *p*-value. All experiments were repeated 3 times. *p < 0.05 vs. CON; **p < 0.001 vs. *CON;* ^*#*^ *p < 0.05* vs. *allopueriol;* ^*#* *#*^ *p < 0.001* vs. *allopueriol;* ^*+*^ *p < 0.05* vs. *H.W;* ^*++*^ *p < 0.001* vs. *H.W;* ^*$*^ *p < 0.05* vs. *20% EtOH;* ^*@*^ *p < 0.05* vs. *40% EtOH;* ^*&*^ *p < 0.05* vs. *60% EtOH;* ^*%*^ *p < 0.05* vs. *80% EtOH;* ^*%%*^ *p < 0.001* vs. *80% EtOH*.

### 3.4 Elastase inhibitory effects of BT extract

**FIGURE 3:**
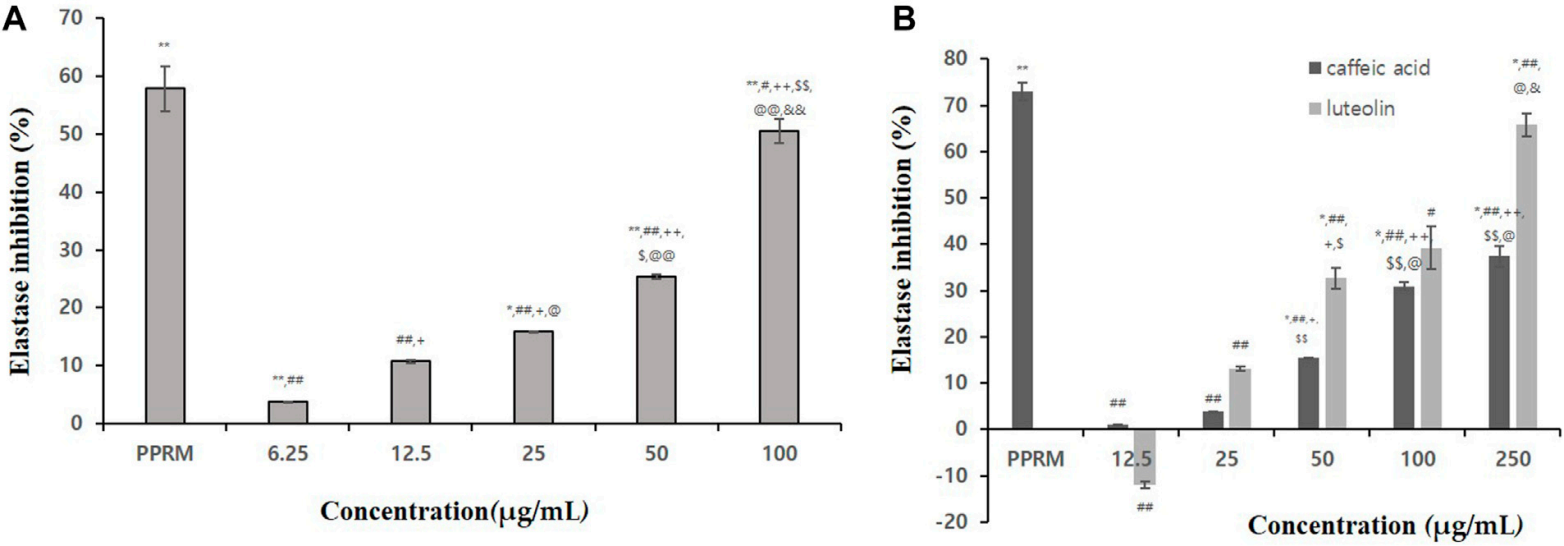
Elastase inhibitory activities of 100% ethanolic extracts **(A)**, caffeic acid and luteolin **(B)**. Statistical significance was expressed as *p*-value. All experiments were repeated 3 times. In Figure 3A *p*-value were expressed as *p < 0.05 vs. CON; **p < 0.001 vs. *CON;* ^*#*^ *p < 0.05* vs. *PPRM;* ^*##*^ *p < 0.001* vs. *PPRM;* ^*+*^ *p < 0.05* vs. *6.25 μg/mL;* ^*++*^ *p < 0.001* vs. *6.25 μg/mL;* ^*$*^ *p < 0.05* vs. *25 μg/mL;* ^*$$*^ *p < 0.05* vs. *25 μg/mL;* ^*@* *@*^ *p < 0.001* vs. *50 μg/mL;* ^*&* *&*^ *p < 0.001* vs. *100 μg/mL*, In Figure 3B, *p*-value were expressed as *p < 0.05 vs. CON; **p < 0.001 vs. *CON;* ^*#*^ *p < 0.05* vs. *PPRM;* ^*##*^ *p < 0.001* vs. *PPRM;* ^*+*^ *p < 0.05* vs. *12.5 μg/mL;* ^*$*^ *p < 0.05* vs. *25 μg/mL;* ^*@*^ *p < 0.05* vs. *50 μg/mL;* ^*&*^ *p < 0.05* vs. *100 μg/mL*.

As a result of confirming the elastase inhibitory effect, phospharamidon (PPRM, a control) at 0.5 mg/mL showed an elastase inhibitory effect of about 84.35%. At a concentration of 1 mg/mL, the extract showed an even elastase inhibitory effect (Table 5). In particular, the effect of 100% ethanol extract was the highest (82.9% inhibition). In Figure 3A, 100% BT extract concentration dependently inhibited the elastase. Caffeic acid and luteolin also inhibited the elastase (37.37% and 65.8% inhibition at 250 μg/mL, respectively, Figure 3B). As a result of confirming the inhibitory effect, 100% ethanolic extract was found to be suitable as an anti-wrinkle plant material.

**TABLE 5:** Elastase inhibition activities of water and ethanolic extracts from *Baccharis trimera.*

Extract	Elastase inhibition (%)
PPRM	84.35 ± 4.115^**^
Hot Water	28.50 ± 0.688^**,##^
20% EtOH	26.18 ± 0.309^**,##,+^
40% EtOH	42.71 ± 0.202^**,##,$^
60% EtOH	52.98 ± 2.894^**,##,$^
80% EtOH	55.85 ± 2.055^**,##,+,$,@^
100% EtOH	82.90 ± 1.147^**,##,+,$$,@@,&,%^

Statistical significance was expressed as *p*-value. All experiments were repeated 3 times. **p < 0.001 vs. *CON;* ^*#* *#*^ *p < 0.001* vs. *PPRM;* ^*+*^ *p < 0.05* vs. *H.W;* ^*$*^ *p < 0.05* vs. *20% EtOH;* ^*$* *$*^ *p < 0.001* vs. *20% EtOH;* ^*@*^ *p < 0.05* vs. *40% EtOH;* ^*@*^ *p < 0.05* vs. *40% EtOH;* ^*&*^ *p < 0.001* vs. *60% EtOH;* ^*%*^ *p < 0.05* vs. *80% EtOH*.

## 4 Discussion

*Baccharis trimera* (BT) is mainly distributed in South America. It has been used in the traditional meditation to improve liver function and inflammatory diseases. Soicke & Leng-Peschlow have reported flavone analysis (quercetin, luteolin, nepetin, eupafolin, apigenin, and hispidulin) contained in BT. Since then, various components and biological activities (antidiabetic, anti-inflammatory, gastroprotective, and hepatoprotective) of BT have been reported (Soicke and Leng-Peschlow, 1987). Report by Soicke & Leng-Peschlow reported that various flavonoids were contained in BT extract. In the BT extract in the present study, luteolin was mainly detected, and caffeic acid was additionally detected. The reason why all flavones in the existing literature were not detected was thought to be due to differences in active ingredients depending on the harvesting season or drying method of BT.

Ximenes et al. have investigated active ingredients of BT and reported quinic acid and quinic acid derivatives. In addition, Ximenes et al. (2022) have applied BT hot water extract to a high fat diet animal model at a dose of 15 mg/kg considering daily intake of adults and derived a significant antidiabetic effect. BT 70% ethanol extract has been reported to inhibit alcoholic liver damage. However, it is only effective at 600 mg/kg in rats, showing limitations in the development of medicinal source (Rabelo et al., 2018).

Chaves, Adami, Acco, Iacomini, & Cordeiro have reported that polysaccharides can be partially purified from hot water extract with a hepatoprotective effect at 100 mg/kg *in vivo*, and the optimal human dose was calculated to be about 487 mg/60 kg haman (Chaves et al., 2020).

Dos Reis Lívero et al., 2016 have reported the gastric protective effect of BT. In 90% ethanol extract, gastric protective factors of ethanol extract were 3,5-O-[E]-dicaffeoylquinic acid (3,5-CQA), 4,5-O -[E]-dicaffeoylquinic acid (4,5-CQA), and 3,4-O-[E]-dicaffeoylquinic acid (3,4-CQA). The gastroprotective effect of BT hot water extract through suppression of gastric acid secretion has also been reported. However, its effective dose was 2 g/kg in mouse, showing limitations as a therapeutic material (Biondo et al., 2011). Paul et al. have reported the anti-inflammatory efficacy of BT. However, I.P. of 400 mg/kg or more was used for rats. An anti-inflammatory effect of TB has been reported. Afer administration, high dosage showed limitations in the application of treatment related to intestinal and intercostal inflammation (Paul et al., 2009).

De Oliveira et al. have obtained a phenol rich fraction and a saponin fraction. After producing BT extracts with ethylaacetate and buthanol, fractions were efficiently separated using a Sephadex column. Among obtained fractions, it was found that the phenol rich fraction was effective for pleurisy inflammation when administered at 15 mg/kg I.P. De Oliveira et al. (2012) have fractionated and partially purified BT for the first time to proceed for the development of a high-purity anti-inflammatory botanical drug. It has been reported that the anti-inflammatory effect of BT is also effective in the saponin fraction of BT extract (Gené et al., 1996).

Uric acid is produced as purine, a highly toxic ammonia-like by-product, is generated as protein is broken down, and uric acid is formed when this purine is detoxified and broken down in the liver. Uric acid is excreted from the body through urine, but excessive consumption of purine-containing foods can lead to excessive production of uric acid (Yang et al., 2022).

In general, high levels of uric acid in the blood are called hyperuricemia, and it refers to a condition in which there is an excessive amount of uric acid in the blood (more than 7.0 mg/dL). If hyperuricemia persists, uric acid crystals may be deposited in the joints, resulting in joint inflammation, or deposited in the skin or kidneys, resulting in diseases such as kidney stones. Because hyperuricemia has no clinical symptoms, it is also called asymptomatic hyperuricemia (Piani et al., 2023). We confirmed that BT extract effectively inhibits xanthine oxidase, the key enzyme that induces hyperuricemia.

The efficacy of BT extract on inflammatory, gastric protection, and liver damage is thought to be due to the existence of various active ingredients such as caffeic acid and derivatives, quinic acid and derivatives, rutin, and luteolin. In the present study, BT hot water and ethanol extracts were prepared. It was confirmed that the 100% ethanolic BT extract had strong xanthine oxidase (XO) and elastase inhibitory activities. Substances mainly identified in 100% ethanolic BT extract were caffeic acid and luteolin. It was confirmed that these substances significantly contributed to the inhibition of xanthine oxidase and elastase.

BT ethanol extract (1 mg/mL) and allopurinol (200 ug/mL) showed equivalent XO activity to. Song and Yoon compared the XO inhibitory ability of *Cudrania tricuspidata* leaf and *Camellia japonica* leaf extracts (1 mg/mL) with allopurinol (200 ug/mL). When the XO inhibitory activity was over 50%, the extract was orally administered in an animal model. XO inhibition in blood and liver is known to show a significant effect (Yoon et al., 2017; Song et al., 2018). Therefore, it is thought that BT ethanol extract with high XO activity can also be used to control hyperuricemia in animal models.

In the present study, 100% BT ethanol extract showed elastase inhibition as well as XO inhibition. There was no report on skin whitening of BT. Results of this study suggest that BT can be easily used as a cosmetic material as well as an oral preparation for traditional medicine. BT 100% ethanol extract is expected to improve skin wrinkles through elastase inhibition. Various functional substances including caffeic acid and luteolin are thought to show elastase inhibition.

BT 100% ethanol extract has lower phenol content and reducing power than hot water and 20–80% ethanol extracts. This might be due to characteristics of individual active substances rather than total antioxidant ability of the extract.

Taken together, our results indicate that 100% ethanolic BT extract has functionality as a material that can reduce gout (hyperuricemia) or gout-induced inflammation and improve skin wrinkles. However, animal/human studies related to its effects on hyperuricemia and skin wrinkle improvement are needed in the future.

## 5 Conclusion

In the present study, component analysis, antioxidant, and xanthine oxidase and elastase inhibitory activities of *B. trimera* (BT) stem extract were evaluated. As a result of confirming the previous report, the exact extraction process of BT extract and physicochemical information such as antioxidant were not clearly described. Therefore, in the present study, hot water and ethanolic extracts were prepared, and the extraction efficiency and antioxidant characteristics of extractd were evaluated.The 80% ethanol extract showed the highest DPPH radical scavenging activity, reducing power, total phenolic contents. The 100% ethanol extract showed the highest xanthine oxidase and elastase inhibitory ability. Caffeic acid, luteolin, o-coumaric acid, palmitic acid, naringenin, protocatechoic acid, and linoleic acid were identified as active substances, however flavone such as quercetin, nepetin, eupafolin, apigenin, and hispidulin or quinic acid derivatives could not be identified. Main xanthine oxidase and elastase inhibitors were caffeic acid and luteolin. 100% ethanolic BT extract contained the highest amounts of caffeic acid and luteolin. It appears to be the main xanthine oxidase, elastase inhibitors. Through this study, we firstly reported the evidence that *B. trimera* (BT) stem extract can be used as functional materials with anti-hyperuricemia and skin disease improving effects. Thus, *B. trimera* (BT) stem extract could be used as an anti-hyperuricemia (gout) natural drug or a cosmetic material.

## Data Availability

The original contributions presented in the study are included in the article/supplementary material, further inquiries can be directed to the corresponding authors.
